# Curcumin alleviates oxidative stress and inhibits apoptosis in diabetic cardiomyopathy via Sirt1‐Foxo1 and PI3K‐Akt signalling pathways

**DOI:** 10.1111/jcmm.15725

**Published:** 2020-09-22

**Authors:** Bin‐cheng Ren, Yu‐fei Zhang, Shan‐shan Liu, Xiao‐jing Cheng, Xin Yang, Xiao‐guang Cui, Xin‐rui Zhao, Hui Zhao, Min‐feng Hao, Meng‐dan Li, Yuan‐yuan Tie, Li Qu, Xue‐yi Li

**Affiliations:** ^1^ Department of Rheumatology and Immunology Second Affiliated Hospital of Xi'an Jiaotong University Xi'an China; ^2^ State Key Laboratory of Crop Stress Biology for Arid Areas and College of Life Sciences Northwest A&F University Yangling China; ^3^ Department of Neurology Xi'an Central Hospital Xi'an China; ^4^ Department of Cardiovascular Medicine Second Affiliated Hospital of Xi'an Jiaotong University Xi'an China

**Keywords:** apoptosis, curcumin, oxidative stress, PI3K‐Akt, Sirt1, type 2 diabetes

## Abstract

Diabetes is a disorder of glucose metabolism, and over 90% are type 2 diabetes. Diabetic cardiomyopathy (DCM) is one of the type 2 diabetes complications, usually accompanied by changes in myocardial structure and function, together with cardiomyocyte apoptosis. Our study investigated the effect of curcumin on regulating oxidative stress (OS) and apoptosis in DCM. In vivo, diabetes was induced in an experimental rat model by streptozoticin (STZ) together with high‐glucose and high‐fat (HG/HF) diet feeding. In vitro, H9c2 cardiomyocytes were cultured with high‐glucose and saturated free fatty acid palmitate. Curcumin was orally or directly administered to rats or cells, respectively. Streptozoticin ‐induced diabetic rats showed metabolism abnormalities and elevated markers of OS (superoxide dismutase [SOD], malondialdehyde [MDA], gp91^phox^, Cyt‐Cyto C), enhanced cell apoptosis (Bax/Bcl‐2, Cleaved caspase‐3, TUNEL‐positive cells), together with reduced Akt phosphorylation and increased Foxo1 acetylation. Curcumin attenuated the myocardial dysfunction, OS and apoptosis in the heart of diabetic rats. Curcumin treatment also enhanced phosphorylation of Akt and inhibited acetylation of Foxo1. These results strongly suggest that apoptosis was increased in the heart of diabetic rats, and curcumin played a role in diabetic cardiomyopathy treatment by modulating the Sirt1‐Foxo1 and PI3K‐Akt pathways.

## 1 INTRODUCTION

Diabetes is one of common endocrine and metabolic disorder diseases, and it leads to multiple complications causing uncountable suffering and incalculable economic losses to worldwide patients.^1^ Globally, the number of people with diabetes mellitus has quadrupled in the past three decades. About 1 in 11 adults now have diabetes mellitus, and 90% of them are type 2 diabetes.^2^ Diabetes has increasingly become a global problem to be solved urgently.

Diabetic patients usually accompanied by metabolic disorders, which directly lead to diabetic cardiomyopathy (DCM).^3^, ^4^ Diabetic cardiomyopathy is characterized by cardiac hypertrophy, changes in ventricular structure, as well as diastolic and systolic dysfunction.^5^ It increases the risk of heart failure developing in diabetic patients,^6^ mostly resulting from cardiomyocyte apoptosis.^7^ One of the most important causes of cardiomyocyte apoptosis is oxidative stress (OS),^8^, ^9^ accompanied by the reduction of antioxidants (such as superoxide dismutase, SOD) and the large production of high‐energy oxidative intermediates (such as reactive oxygen species, ROS), thus increase the apoptosis of cardiomyocyte.^10^, ^11^

Curcumin is a natural polyphenol isolated from the root of *Curcuma longa*, and it has antioxidant, anti‐inflammatory, anti‐apoptosis and anti‐carcinogenic properties.^12^, ^13^ Recent research revealed a powerful role for curcumin including alleviating myocardial apoptosis and improving cardiac function in experimental diabetic rats.^14^ Curcumin participates in regulation by involvement in a complex molecular regulatory network, including protein kinase C, c‐Jun N‐terminal kinase (JNK) and advanced glycation end‐product pathways.^14^, ^15^ In vitro, curcumin can prevent OS induced by NADPH oxidase (NOX) and inhibit cardiomyocyte apoptosis induced by high glucose.^16^ ROS produced by NOX plays an important role in controlling metabolism and regulating insulin secretion under physiological conditions. Increased ROS levels in abnormal conditions such as HG/HF intake may induce apoptosis via JNK, PI3K‐Akt and other signalling pathways.

Sirtuin 1 (Sirt1) is a highly conserved nicotinamide adenosine dinucleotide (NAD) ‐dependent deacetylase, which plays regulatory role in metabolism and ageing. Sirt1 can deacetylate histones and several transcription regulators in the nucleus, as well as specific proteins in the cytoplasm and mitochondria, including Sirt1 inhibiting transcription factors (NF‐κB, MMP‐9, FOXO3a and p53), eNOS, PGC‐1α and AMPK.^17^ Sirt1 regulates the metabolism of fat and glucose by deacetylating the target proteins, thus acting as crucial regulator of cellular anti‐stress, energy metabolism and tumorigenesis, delaying the onset of age‐related disease and extending a healthy lifespan.^18^ In vivo, emerging evidence from mouse models indicates Sirt1 is a potent protector from ageing‐associated pathologies, such as cancer, liver steatosis, neurodegeneration, cardiovascular disease and diabetes.^19^

However, there are still a few details that remain unclear, such as the mechanism of curcumin in inhibiting apoptosis and the relationship among multiple signalling pathways mentioned above. Our study investigated the effect of curcumin on regulating apoptosis in DCM.

## 2 MATERIALS AND METHODS

### 2.1 Animals

The experiments were performed in accordance with the Guide for the Care and Use of Laboratory Animals published by the US National Institutes of Health (NIH Publication No. 8523, revised 1996) and with approval from the Second Affiliated Hospital of Xi'an Jiaotong University. We purchased 45 male Sprague‐Dawley rats at age 4 weeks from Xi'an Jiaotong University. After being fed a high‐glucose and high‐fat diet (containing 40% fat, 41% carbohydrates and 18% protein) for 4 weeks, rats for diabetic model were induced by intraperitoneal injection of streptozotocin (STZ [Biochem Partner, Shanghai], 60 mg/kg, i.p.) for 3 consecutive days. Streptozotocin was freshly prepared in citrate buffer (0.1 mmol/L, pH 4.2‐4.5). Rats in control group (n = 10) were injected with citrate buffer alone after being fed a 4‐week basic diet. One week after STZ injection, oral glucose tolerance test (OGTT) was performed to confirm STZ injection‐induced hyperglycaemia. Rats were not allowed to eat after 10 PM to make a fasting state. The next morning, rats were orally administrated by 75 g of liquid glucose, and blood glucose (in mmol/L) from tail vein blood samples were measured with a blood glucose meter at 30, 60, 90 and 120 minutes later. Rats with high blood glucose levels (≥13.5 mmol/L) and poor glucose tolerance were considered as diabetic rats. Body weight and blood glucose of rats were recorded every week during the whole experiment. Diabetic model (DM) rats (5 died) were randomly divided into two groups (15 in each group). Curcumin (purchased from Biochem Partner) was orally administered by drinking water (100 mg/kg/d) to treated DM rats (DM + Cur). Untreated DM rats and control rats (Con) received a 1% carboxyl methyl cellulose‐Na solution. All rats were sacrificed at age 12 weeks.

### 2.2 Echocardiography

After measurement of blood glucose and body weight, 2‐D and M‐mode echocardiography were performed by blinded operators with a Vevo 770 High‐Resolution Imaging System (Visual Sonics, Toronto, ON, Canada) to assess cardiac function. Rats were anaesthetized by inhaling 3% isoflurane (ISO), then placed horizontally on the table, maintaining a low dose (1.5%‐2%) of ISO for continuous anaesthesia. The echocardiography parameters involved heart rate (HR), interventricular septum thickness (IVST), left ventricular end diastolic volume (LVEDV), left ventricular end systolic volume (LVESV), left ventricular ejection fraction (LVEF) and left ventricular fractional shortening (LVFS). All measurements represented the mean of five consecutive cardiac cycles.

### 2.3 Histopathology

After measurement of heart weight, half rats of each group were used for histochemistry experiments. The entire rat hearts were immersed in 4% paraformaldehyde and placed in treatment boxes. After a series of gradient alcohol dehydration, they were embedded in paraffin blocks. Paraffin tissues were cut into 4‐μm‐thick sections, dewaxed in xylene, rehydrated by reducing ethanol concentration, washed in phosphate‐buffered saline, then stained with Masson trichrome stain. After staining, the slides were dehydrated by gradient concentration of ethanol and xylene. Photographs were taken under an optical microscope (original magnification 3400; Nikon), and histopathological damage was evaluated. 4′, 6‐diamino‐2‐phenylindole (DAPI) was obtained from Solarbio Technology (Beijing).

### 2.4 Cell culture and treatment

H9c2 cardiomyocytes were purchased from Shanghai Cellular Research Institute (Shanghai) and cultured in Dulbecco's Modified Essential Medium (DMEM, Hyclone, USA) containing 10% foetal bovine serum (Hyclone, USA) and 1% penicillin‐streptomycin (Hyclone, USA) at 37°C, humidified 95% air and 5% CO_2_. In the curcumin treatment experiment, cells were divided into control group (Con) with 5.5 mmol/L glucose, mannitol group (Mannitol) with 5.5 mmol/L mannitol, high‐glucose and high‐fat group (HG/HF) with 25 mmol/L glucose and saturated free fatty acid palmitate (500 μmol/L), and HG/HF with curcumin group (HG/HF + Cur). In the inhibitor experiment, cells were divided into control group, HG/HF group, HG/HF with EX527 group (HG/HF + EX527), HG/HF with curcumin and EX527 group (HG/HF + Cur+EX527), HG/HF with LY294002 group (HG/HF + LY294002), and HG/HF with curcumin and LY294002 group (HG/HF + Cur+LY294002). Both two inhibitors were from Medchem Express (NJ, USA). Before treatment, cells of each group were cultured in low‐glucose medium overnight (12 hours), and then replaced with new low‐glucose medium. All groups were collected for further examination after 10‐hr treatment.

### 2.5 Assessment of cell viability

Cell viability was assessed by 3‐(4, 5‐dimethyl‐2‐thiazolyl)‐2, 5‐diphenyl‐2‐H‐tetrazolium bromide (MTT) assay. H9c2 cells were cultured in DMEM containing 10% foetal bovine serum for 24 hours in 96‐well culture plates at 1 × 10^4^ cell per well. Cells were divided into groups of Con, Mannitol, HG/HF, Con with curcumin, HG/HF with curcumin, Con with two inhibitors, as well as combinations of HG/HF, curcumin, inhibitors. MTT was added at a final concentration of 0.5 mg/mL for 4 hours Thereafter, 100 μL DMSO was added to each well and absorbance was measured by using a microplate reader (Thermo Fisher Scientific, USA) with wavelength 490 nm. Cell viability is described as the ratio of the optical density of samples to that of the control.

### 2.6 TUNEL assay

Apoptosis of tissue and cell was assessed by TUNEL assay. Apoptotic detection kit (Roche) was used to detect DNA fragments in the nucleus in situ. TUNEL‐positive cells and total cells were counted, while the ratio was calculated.

### 2.7 Quantification of SOD and MDA

Hearts or cells were collected and homogenized in phosphate‐buffered saline. The amount of SOD and MDA was measured by Superoxide Dismutase (SOD) assay kit and Malondialdehyde (MDA) assay kit (Nanjing jiancheng Biotech, Nanjing, China).

### 2.8 Western blot analysis

Total protein was separated from hearts or cells, separated by SDS‐PAGE, and transferred to PVDF membranes. After being blocked with 5% non‐fat dry milk at room temperature (RT) for 2 hours, the protein was incubated with antibodies against Cleaved caspase‐3 (1:1000), caspase‐3 (1:1000), Bcl‐2 (1:1000), Bax (1:1000), gp91^phox^ (1:500), Cyt‐Cyto C (1:1000), NQO1 (1:1000), Nrf2 (1:1000), Sirt1 (1:1000), Ac‐Foxo1 (1:500), Foxo1 (1:1000), PI3K (1:1000), p‐Akt (1:1000), Akt (1:1000) and β‐Actin (1:1000) at 4°C for 14 hours. After washed with TBST buffer (50 mmol\L Tris‐HCl, 150 mmol\L NaCl, 0.1% Tween 20, pH 7.6) for three times, the membranes were incubated with horseradish peroxidase‐conjugated secondary antibodies (1:5000) at room temperature for 2 hours. Immunocomplexes were detected with an enhanced chemiluminescent protein detection kit, and bands were quantified by scanning densitometry with an image analyzer (BandScan, USA). Primary antibodies against Sirt1, PI3K, p‐Akt, Akt, Cleaved caspase‐3, caspase‐3, Bcl‐2 and Bax were from Cell Signaling Technology (Boston, MA, USA); antibodies against gp91^phox^ and β‐actin were from Santa Cruz Biotechnology; Ac‐Foxo1 and Foxo1 were from Medchem Express (NJ, USA). Secondary antibodies, including rabbit anti‐goat, goat anti‐mouse and goat anti‐rabbit antibodies, were from Zhongshan Co. (Beijing).

### 2.9 Statistical analysis

Statistical analyses were performed using GraphPad Prism v5.0 (Graph Pad Software, San Diego, CA, USA). All data are presented as mean ± SEM. One‐way ANOVA was used to assess the differences between the groups, and then post hoc testing was conducted through Bonferroni correction as appropriate. *P* < 0.05 was considered statistically significant.

## 3 RESULTS

### 3.1 Curcumin improves cardiac function in STZ‐induced type 2 diabetes rats

**FIGURE 1:**
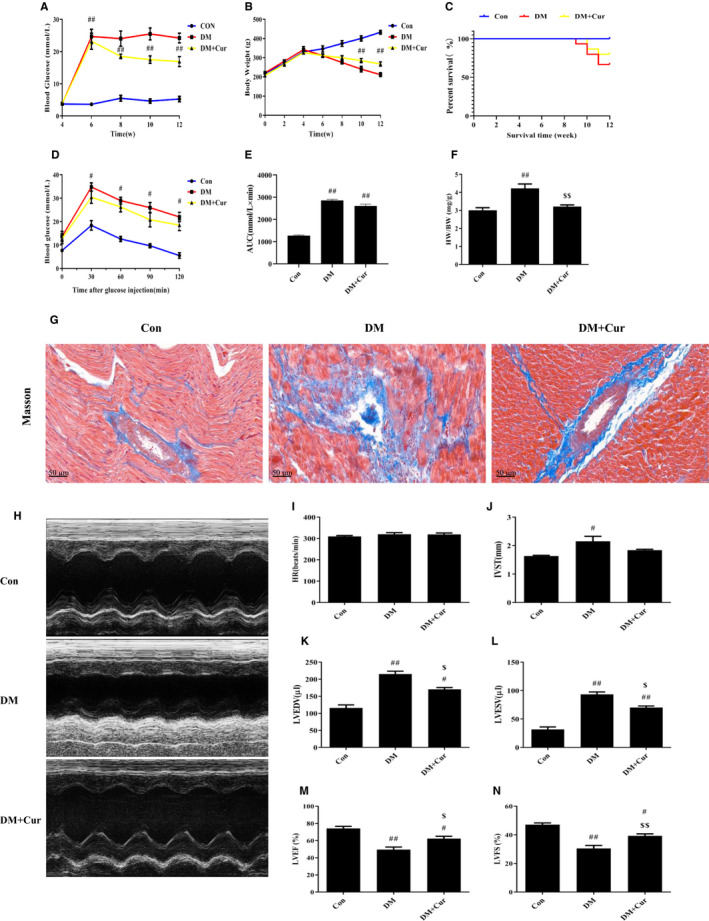
Rats were treated and measured in different groups. The results of blood glucose (A), body weight (B), survival curve (C) and oral glucose tolerance test (D) in different groups were showed in different colours: Con (blue), DM (red), DM + Cur (yellow). (E) area under the curve (AUC) (mmol/L × min). (F) Heart‐to‐body weight ratio (HW/BW) (mg/g). (G) Masson trichrome stain of collagen deposition around the cardiovascular. (H‐N) Cardiac function including heart rate (HR) (beats/min), interventricular septum thickness (IVST) (mm), left ventricular end diastolic volume (LVEDV) (μL), left ventricular end systolic volume (LVESV) (μL), left ventricular ejection fraction (LVEF) (%), left short‐axis fractional shortening (LVFS) (%) and M‐mode images by echocardiographic detection. ^#^ *P* < 0.05, ^##^ *P* < 0.01 compared with Con; ^$^ *P* < 0.05, ^$$^ *P* < 0.01 compared with DM

STZ‐induced DM rats showed typical characteristics of type 2 diabetes, including increased blood glucose and decreased body weight (Figure 1A,B, *P* < 0.01 vs. Con), while curcumin treatment improved these features. There is no significant difference in survival time of different groups (Figure 1C). Moreover, the glucose tolerance status of DM and DM + Cur rats on OGTT is worse than that of control rats (Figure 1D, *P* < 0.05 vs. Con), and the analysis of the area under the curve (AUC) also reflected this change (Figure 1E, *P* < 0.01 vs Con). In addition, an increase in the heart‐to‐weight ratio (HW/BW) of DM rats (Figure 1F, *P* < 0.01) indicated that the heart and cardiac function might be affected. Masson trichrome stain of cardiac section showed excessive collagen deposition around the cardiovascular in the DM group, indicating that cardiac remodelling occurred, while the DM + Cur group recovered (Figure 1G). The results of the echocardiographic detection of cardiac function showed no difference in HR between the three groups (Figure 1I). The increased IVST (*P* < 0.05), LVEDV (*P* < 0.01), LVESV (*P* < 0.01) and the decreased LVEF (*P* < 0.01), LVFS (*P* < 0.01) indicated damaged cardiac pumping capacity of DM rats (Figure 1H‐N). In contrast, cardiac function was markedly improved in curcumin‐treated rats (Figure 1H‐N, *P* < 0.05 vs. DM). Therefore, curcumin could improve STZ‐induced decrease in cardiac construction and function of DM rats.

### 3.2 Curcumin alleviates oxidative stress and inhibits apoptosis of cardiomyocytes in type 2 diabetes rats

**FIGURE 2:**
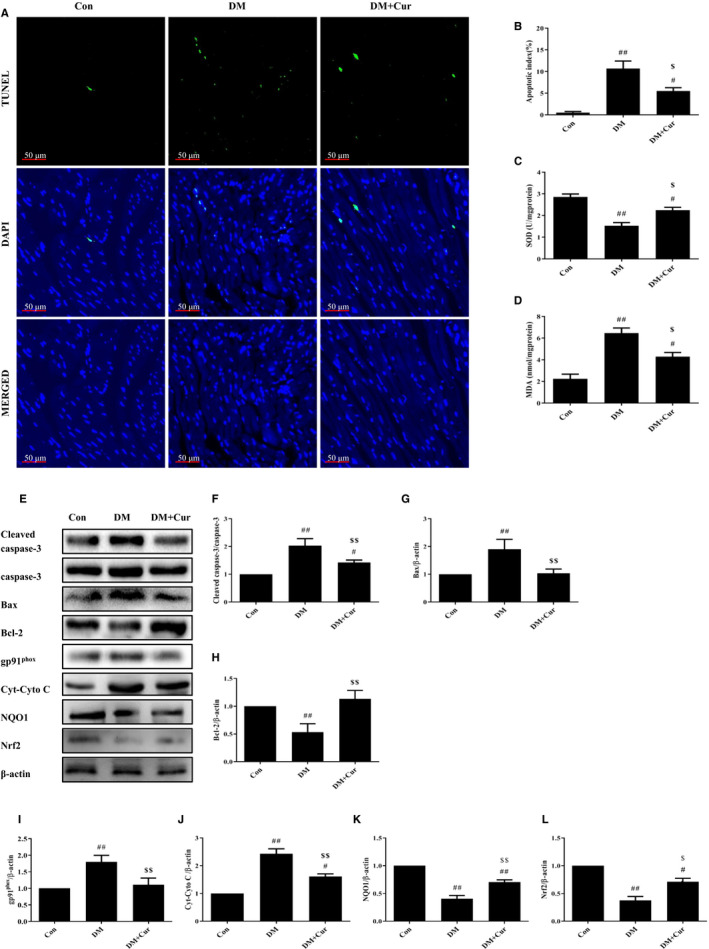
Curcumin mitigates OS and apoptosis in rats. (A‐B) Immunofluorescence staining of TUNEL‐positive cells and apoptosis index in Con, DM, and DM + Cur. (C‐D) Measurement of OS‐related markers level (SOD and MDA). (E‐L) Western blot analysis of protein markers related to OS and apoptosis. ^#^ *P* < 0.05, ^##^ *P* < 0.01 compared with Con; ^$^ *P* < 0.05, ^$$^ *P* < 0.01 compared with DM

TUNEL‐positive cardiomyocytes in DM were more numerous (Figure 2A,B, *P* < 0.01), while in DM + Cur was much less (*P* < 0.05 vs DM). Western blot analysis revealed that the ratio of Cleaved caspase‐3/caspase‐3 and the expression of Bax in DM were higher, while the expression of Bcl‐2 was lower (Figure 2E‐H, *P* < 0.01), indicating severe apoptosis. In DM + Cur, the expression of the above markers basically returned to normal, and the apoptosis was relieved (*P* < 0.01 vs. DM).

Consistently, the activity of SOD in cardiomyocytes of DM was lower and the amount of MDA was higher (Figure 2C,D, *P* < 0.01). In DM + Cur, the activity of SOD increased and the amount of MDA reduced (*P* < 0.05 vs. DM). Moreover, the expression of gp91^phox^ and Cyto C in DM was higher, while the expression of NQO1 and Nrf2 was decreased. The expression of these markers was significantly reversed in DM + Cur (Figure 2E,I‐L, *P* < 0.01 vs. DM). These results suggested increased cardiomyocyte apoptosis in STZ‐induced diabetes rats, which could result from OS. Curcumin could effectively relieve OS and inhibit apoptosis in cardiomyocytes of type 2 diabetes rats.

### 3.3 Curcumin inhibits apoptosis via Sirt1‐Foxo1 and PI3K‐Akt signalling in type 2 diabetes rats

**FIGURE 3:**
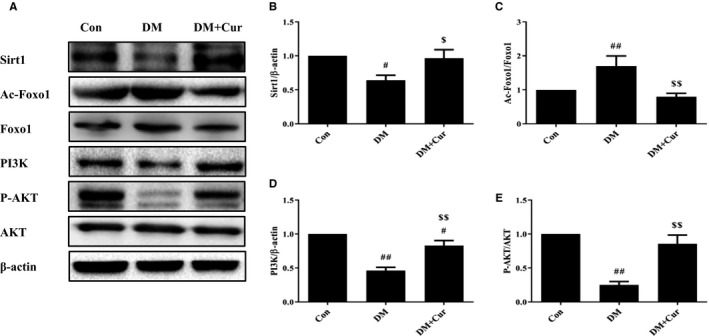
Curcumin acts via Sirt1‐Foxo1 and PI3K‐Akt signalling pathways in rats. (A‐E) Western blot analysis of Sirt1, PI3K, the ratio of p‐Akt/Akt, and the ratio of Ac‐Foxo1/Foxo1. ^#^ *P* < 0.05, ^##^ *P* < 0.01 compared with Con; ^$^ *P* < 0.05, ^$$^ *P* < 0.01 compared with DM

For further study of the molecular mechanism, we found that the protein expression levels of Sirt1, PI3K and the phosphorylation of Akt in DM cardiomyocytes decreased significantly, while the acetylation of Foxo1 increased. Curcumin treatment could almost restore to control levels (Figure 3A‐E). These results suggested that both Sirt1‐Foxo1 and PI3K‐Akt signalling pathways were inhibited in DM and could be strengthened by curcumin treatment. Curcumin maybe prevent cardiomyocyte apoptosis via Sirt1‐Foxo1 and PI3K‐Akt signalling pathways.

### 3.4 Curcumin inhibits apoptosis of H9c2 cardiomyocytes via Sirt1‐Foxo1 and PI3K‐Akt signalling pathways in HG/HF situation

To corroborate the effect of curcumin on apoptosis in vitro, H9c2 cardiomyocytes were exposed to HG/HF and treated with gradient concentrations of curcumin. Mannitol was used as a control to exclude the effect of HG/HF‐induced hyperosmolality on cells.

**FIGURE 4:**
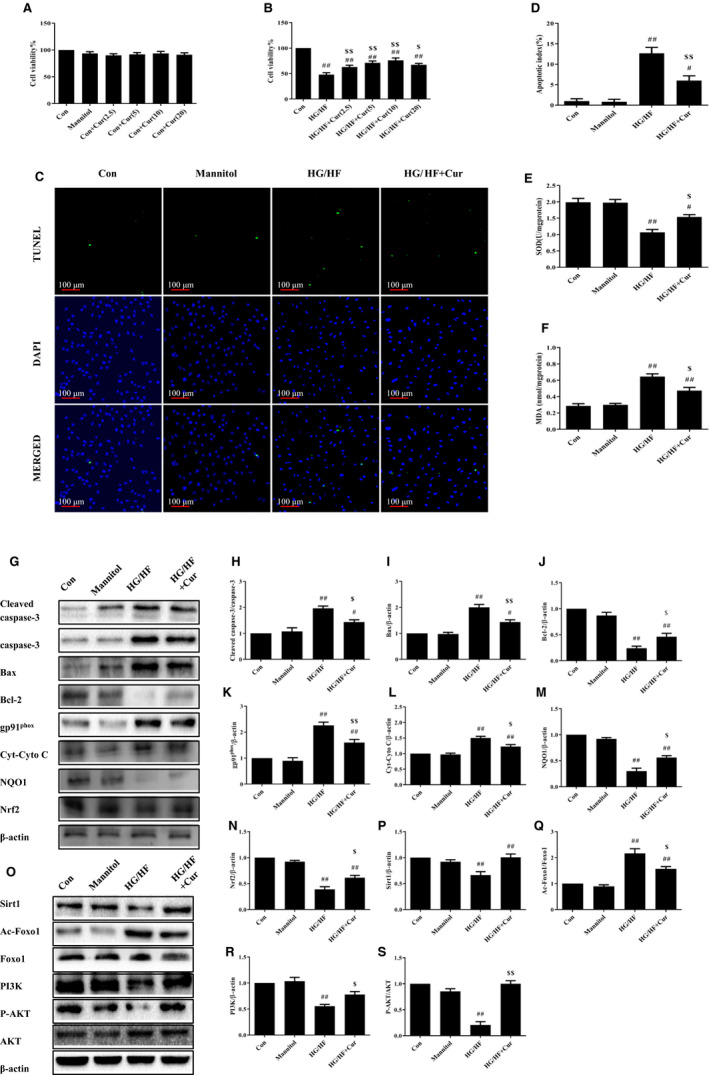
Curcumin mitigates OS and apoptosis of H9c2 cells in HG/HF. Cell viability of H9c2 cells with (A) mannitol and gradient concentration curcumin; (B) HG/HF and gradient concentration curcumin by MTT. (C‐D) Immunofluorescence staining of TUNEL‐positive cells and apoptosis index in Con, Mannitol, HG/HF, and HG/HF + Cur. (E‐F) Measurement of SOD and MDA levels in H9c2 cells. (G‐N) Western blot analysis of protein markers related to apoptosis and to OS. (O‐S) Western blot analysis of Sirt1, PI3K, the ratio of p‐Akt/Akt, and the ratio of Ac‐Foxo1/Foxo1. ^#^ *P* < 0.05, ^##^ *P* < 0.01 compared with Con; ^$^ *P* < 0.05, ^$$^ *P* < 0.01 compared with HG/HF

Curcumin alone or hyperosmolality had no effect on cell viability (Figure 4A). Once again, the cell viability of HG/HF cells was greatly decreased, and all doses of curcumin could effectively improve viability (Figure 4B, *P* < 0.01).

In addition, the proportion of TUNEL‐positive cells in HG/HF was much higher than that in Con, while the proportion in HG/HF + Cur was significantly lower than that of HG/HF (Figure 4C,D). Moreover, the activity of SOD decreased in HG/HF and the amount of MDA increased, indicating severe OS. These were effectively alleviated in HG/HF + Cur (Figure 4E,F). Western blot results of cardiomyocytes showed the same results as in vivo. The ratio of Cleaved caspase‐3/caspase‐3 and the expression of Bax in HG/HF increased, while the expression of Bcl‐2 decreased (*P* < 0.01). The expression of gp91^phox^ and Cyto C in HG/HF was increased and that of NQO1 and Nrf2 was decreased, which indicated increased apoptosis. Curcumin relieved apoptosis in H9c2 cardiomyocytes (Figure 4G‐N).

Sirt1‐Foxo1 and PI3K‐Akt pathways were inhibited in HG/HF. The expression of Sirt1, PI3K and the ratio of p‐Akt/Akt was reduced significantly, and the ratio of Ac‐Foxo1/Foxo1 was increased. In HG/HF + Cur, the expression of Sirt1 and PI3K was activated, together with the recovery of the ratio of p‐Akt/Akt (Figure 4O‐S).

In the above experiments, we found no significant difference between mannitol and the control condition, so curcumin itself contributed to the effect of preventing apoptosis. These results further prove that curcumin can effectively protect the H9c2 cardiomyocytes from OS‐induced apoptosis in diabetic rats, whereas Sirt1‐Foxo1 and PI3K‐Akt signalling pathways were involved in the regulation.

### 3.5 Inhibitors of Sirt1‐Foxo1 and PI3K‐Akt signalling pathways blocked the rescue of curcumin

To further confirm that curcumin could inhibit cardiomyocyte apoptosis via Sirt1‐Foxo1 and PI3K‐Akt pathways, we used specific inhibitors EX527 (Sirt1 inhibitor) and LY294002 (PI3K inhibitor) to block these two pathways.

**FIGURE 5:**
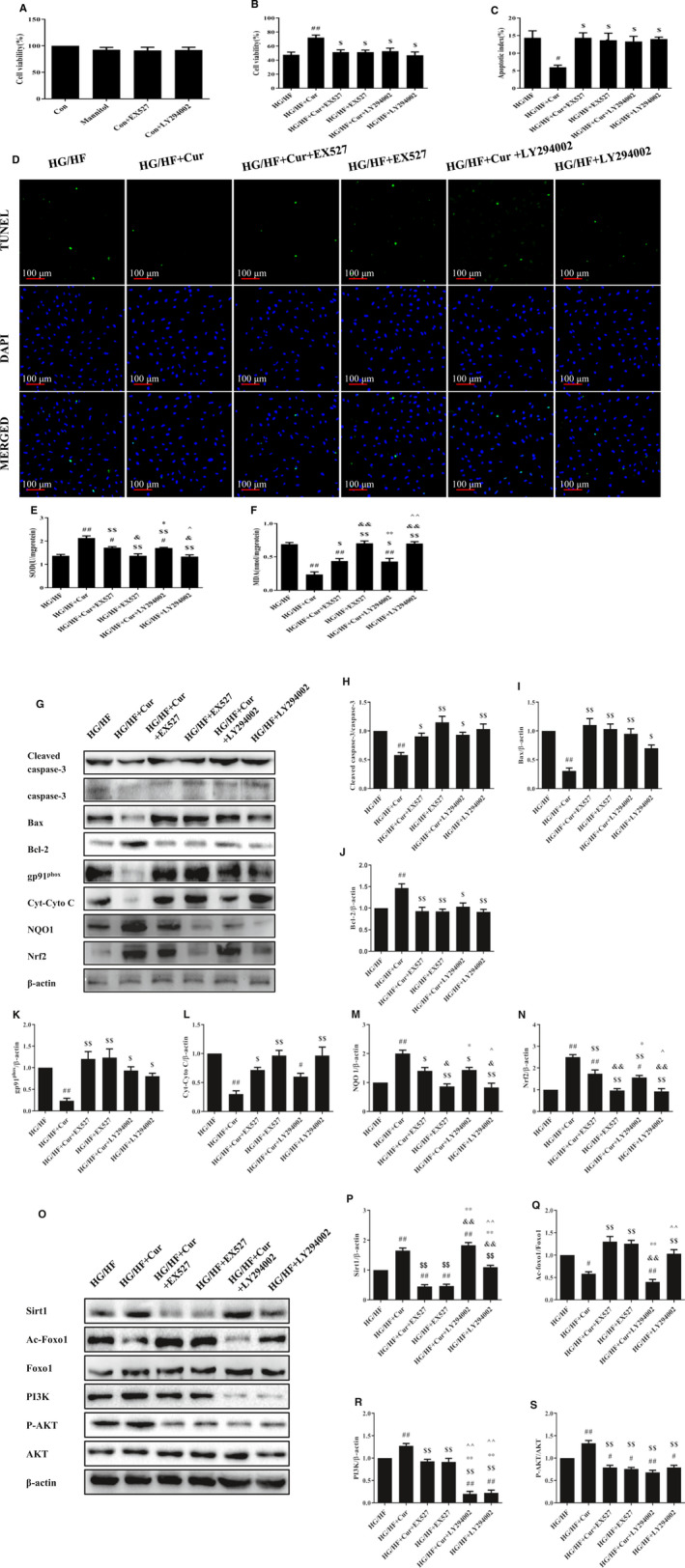
Inhibitors of Sirt1‐Foxo1 and PI3K‐Akt signalling pathways blocked the rescue of curcumin. The cell viability of H9c2 cells with (A) mannitol and two inhibitors; (B) HG/HF with curcumin and two inhibitors by MTT. (C‐D) Immunofluorescence staining of TUNEL‐positive cells and apoptosis index in Con, HG/HF, HG/HF + EX527, HG/HF + Cur+EX527, HG/HF + LY294002, and HG/HF + Cur+LY294002. (E‐F) Measurement of SOD and MDA levels in H9c2 cells. (G‐J) Western blot analysis of protein markers related to apoptosis. (K‐N) Western blot analysis of protein markers related to OS. (O‐S) Western blot analysis of Sirt1, PI3K, the ratio of p‐Akt/Akt, and the ratio of Ac‐Foxo1/Foxo1. ^#^ *P* < 0.05, ^##^ *P* < 0.01 compared with Con; ^$^ *P* < 0.05, ^$$^ *P* < 0.01 compared with HG/HF; ^&&^ *P* < .01, compared with HG/HF + Cur+EX527; ^*^ *P* < 0.05, ^**^ *P* < 0.01, compared with HG/HF + Cur+LY294002; ^*P* < 0.05, ^^*P* < 0.01, compared with HG/HF + LY294002

The hyperosmolality effect of inhibitors was eliminated by comparing Con and Mannitol (Figure 5A). Regardless of the presence of curcumin, the cell viability of all groups treated with HG/HF and EX527 or LY294002 decreased (Figure 5B). In addition, the proportion of TUNEL‐positive cells decreased in HG/HF + Cur, while groups with inhibitors did not change compared with HG/HF, regardless of the presence or absence of curcumin (Figure 5C,D). Curcumin could increase the activity of SOD in HG/HF and reduce the amount of MDA (*P* < 0.01), but these effects of rescue were blocked by two inhibitors (*P* < 0.05). Only when both Sirt1‐Foxo1 and PI3K‐Akt pathways were activated and curcumin was present, cell viability could be improved and OS/apoptosis be alleviated (Figure 5E,F).

Consistently, Western blot analysis revealed that in the HG/HF group and HG/HF with inhibitor groups, the ratio of Cleaved caspase‐3/caspase‐3 and the expression of Bax were higher, while the expression of Bcl‐2 was lower, indicating more severe apoptosis. Although curcumin was inhibited, the ratio of Cleaved caspase‐3/caspase‐3 with curcumin treatment was slightly lower than that with EX527 or LY294002 treatment (Figure 5G‐J). In addition, curcumin treatment could still restore the expression of OS‐related proteins gp91phox, Cyto C, NQO1 and Nrf2 to a certain extent in the presence of inhibitors in HG/HF cardiomyocyte. Inhibitors did restrain curcumin effect but could not completely prevent it (Figure 5K‐N).

In addition, the expression of Sirt1 and PI3K was significantly reduced by EX527 and LY294002, respectively, and the two pathways were blocked (Figure 5O‐S). Curcumin increased the ratio of p‐Akt/Akt, while inhibitor treatment decreased the ratio of p‐Akt/Akt significantly, even lower than HG/HF. Importantly, after inhibiting the expression of Sirt1, curcumin almost lost the ability to inhibit Foxo1 and promote the expression of Sirt1 and PI3K, but not after inhibiting the expression of PI3K. Curcumin directly promoted Sirt1 and inhibited Foxo1, which could not be affected by blocking PI3K. These results demonstrate that PI3K could be downstream of Sirt1, and both factors could respond to curcumin. Curcumin could directly regulate Sirt1, and PI3K might be located downstream of Sirt1, so blocking PI3K did not affect the inhibition of Foxo1 and the promotion of Sirt1 by curcumin. Therefore, curcumin could activate Sirt1‐Foxo1 and PI3K‐Akt pathways, thereby reducing OS and inhibiting apoptosis.

## 4 DISCUSSION

Diabetes is one of the most common non‐communicable disease that threaten human health and life.^20^ In our study, we used STZ‐induced HG/HF fed rats as a type 2 diabetes model. The model rats showed metabolism abnormalities, changes in ventricular structure, and elevated OS and apoptosis markers. Secondly, curcumin attenuated myocardial dysfunction of diabetic rats in vivo. Thirdly, curcumin protected against OS damage and cardiomyocyte apoptosis caused by type 2 diabetes in vivo and in vitro. Fourthly, curcumin also enhanced Akt phosphorylation and inhibited Foxo1 acetylation, indicating that it might reduce OS damage of type 2 diabetes and protect cardiomyocyte from apoptosis by modulating Sirt1‐Foxo1and PI3K‐Akt pathways.

In previous research, the combination of STZ induction with HG/HF intake was considered a general strategy for obtaining an animal model of the type 2 diabetes because it causes increased blood glucose, weight loss and a positive OGTT test, thus simulating the real course of human type 2 diabetes mellitus.^21^, ^22^ Our test results indicated that the modelling was successful, and the method of STZ‐induced type 2 diabetic rats was feasible. Type 2 diabetes has been shown to cause decreased cardiac function and disordered arrangement of cardiomyocytes, heart failure and even life‐threatening.^23^, ^24^ Essentially, heart failure is due to type 2 diabetes leading to OS damage and cardiomyocyte apoptosis.^25^, ^26^ Our results confirmed these conclusions.

Curcumin has been previously shown to exert antioxidant, anti‐inflammatory properties and anti‐carcinogenic activity, which has attracted the attention of researchers.^27^, ^28^ We found that curcumin could significantly improve diabetic cardiomyopathy by reducing OS and apoptosis in cardiomyocytes. Firstly, curcumin eliminated elevated blood glucose and delayed the onset of cardiovascular complications by controlling metabolic abnormalities.^29^ Our results confirmed that curcumin was involved in the control of glycemic metabolism.

Secondly, curcumin ameliorated cardiac dysfunction in diabetic rats. In the development of diabetic cardiomyopathy, cardiomyocyte death and limited regeneration of cardiomyocytes usually lead to pathological changes, which ultimately damage the structure and function of cardiomyocytes. In vivo, echocardiographic examination results of cardiac function and Masson staining results of cardiomyocyte tissue sections indicated that curcumin improved cardiac dysfunction in diabetic cardiomyopathy.

Finally, curcumin attenuated OS and cardiomyocyte apoptosis. OS is defined as the imbalance between the production and elimination of free radicals, which plays a critical role in the development of heart failure and left ventricular remodelling in DCM.^30^ Studies demonstrated that MnSOD can increase the activity of endothelial nitric oxide synthase (eNOS), reduce the loading of superoxide anions and increase the survival rate under oxidative stress.^31^ Besides, NADPH oxidase (NOX) is a multi‐subunit complex consisting of a family of molecules with a series of homologs and gp91^phox^ is a major NOX isoform expressed in cardiomyocytes.^32^, ^33^ NOX is a critical determinant of myocardial ROS generation^34^; and the activation of NOX occurs via multiple signalling pathways.^35^ In this study, curcumin restored the activity of SOD in the heart of diabetic rats and decreased the expression of gp91^phox^. Bcl‐2 is an antiapoptotic protein, it inhibits apoptosis by inhibiting Bax/Bak oligomerization to increase mitochondrial membrane permeability and prevent Cyto C release. The expression of Bcl‐2 was reduced under the diabetic stimulus and recovered by the addition of curcumin in the present study.

Furthermore, curcumin activated Sirt1‐Foxo1 and PI3K‐Akt signalling pathways in the heart of diabetic rats. Several studies have pointed out that Sirt1 has a cardioprotective effect, and its expression in the heart is down‐regulated by many stress stimuli that collectively drive the pathogenesis of myocardial infarction.^36^ In our data, the expression of Sirt1 reduced in the heart of DM rats and HG/HF H9c2 cells, and recovered after curcumin treatment. Stimulation of the cytosolic Sirt1 has cardioprotective effects on ischaemia‐reperfusion injury.^37^ Sirt1/PGC‐1α signalling pathway can enhance mitochondrial biogenesis, thereby protecting acute heart failure rats.^38^ In addition, the enhanced Sirt1/PGC‐1α signalling pathway can play a role in calorie restriction (CR), thus reducing OS, fibrosis, inflammation and protecting the heart.^39^ Sirt1 can regulate inflammation, ageing (life span and health span) through deacetylation of transcription factors and histones. In chronic inflammatory conditions and ageing, the level and activity of Sirt1 are reduced, and OS is increased.^40^ Besides, Sirt1 is a positive regulator of insulin secretion, which can trigger the absorption and utilization of glucose, and at the same time reduce the high‐glucose concentration of blood by regulating various metabolic pathways.^41^ Sirt1 can greatly attenuate islet α cell hyperplasia in diabetic mice, reduce plasma glucagon concentration, significantly improve blood glucose control and thus treat type 1 diabetes.^42^

Akt has a well‐defined role in regulating cardiovascular function (such as heart growth, myocardial contractile function and coronary angiogenesis) and can promote cell survival by inhibiting multiple targets in the apoptosis signalling cascade. Studies have shown that PI3K/Akt signalling pathway is involved in the growth, metabolism and apoptosis of myocardial cells, protecting the heart through PIK3CA.^43^ Additionally, the PI3K‐Akt‐FoxO3a pathway can prevent STZ‐induced cardiac function deterioration and structural cardiomyopathy in diabetic rats and reduce the apoptosis of diabetic cardiomyocytes.^44^ The decrease of AKT‐Foxo1 phosphorylation level is closely related to the occurrence of insulin resistance and apoptosis in DCM, and it plays an important role in inhibiting apoptosis and improving the cardiac function of DCM.^45^ We reported for the first time that curcumin can play a protective role by activating the Sirt1‐Fox1 and PI3K‐Akt pathways of type 2 diabetic cardiomyopathy. And for the first time, Sirt1 and PI3K‐Akt are directly connected together. Experiments using pathway inhibitors demonstrated that Sirt1 is upstream of PI3K‐Akt.

The pathogenesis of DCM is complicated. It is currently known that Sirt1‐Fox1, PI3K‐Akt and other signalling pathways can inhibit OS and apoptosis in cardiomyocytes, thereby achieving the purpose of remission and treatment of DCM. The deficiency is that the evidence on the relationship between these two pathways is slightly weak, and further research is needed.

Our results demonstrate that Sirt1‐Foxo1 and PI3K‐Akt pathways are involved in mediating the effect of curcumin on DCM. Curcumin activates the Sirt1‐Foxo1 pathway and PI3K‐Akt survival pathway, blocks Foxo1 accumulation and eliminates ROS levels. In untreated diabetic hearts, gp91^phox^ accumulates in myocardium, SOD activity decreases, Sirt1‐Foxo1 and PI3K‐Akt pathways are inactivated, and eventually apoptosis occurs. Our research strongly suggests that curcumin may have therapeutic potential in the treatment of DCM and other cardiovascular diseases by improving metabolic abnormalities, OS and cardiomyocyte apoptosis pathways.

## AUTHOR CONTRIBUTIONS

Xue‐yi Li: Conceiving and designing the experiments. Bin‐cheng Ren and Yu‐fei Zhang: Performing the experiments. Shan‐shan Liu, Xiao‐jing Cheng: Analysing the data. Xin Yang, Xiao‐guang Cui, Xin‐rui Zhao, Hui Zhao, Min‐feng Hao, Meng‐dan Li, Yuan‐yuan Tie, Li Qu: Contribution to reagents/materials/analysis tools. Bin‐cheng Ren and Yu‐fei Zhang: Writing the paper.

## ETHICAL APPROVAL AND CONSENT TO PARTICIPATE

All experimental procedures for animals were approved by the Animal Ethics Committee of the Second Affiliated Hospital, Xi'an Jiaotong University in accordance with the National Institutes of Health Guide for Care and Use of Laboratory Animals (NIH Publications, No. 8023, revised 1978).

## Data Availability

All data generated or analysed during this study are included in this published article.
